# Derivation of unifying formulae for convective heat transfer in compressible flow fields

**DOI:** 10.1038/s41598-021-95810-0

**Published:** 2021-08-18

**Authors:** Bo Zhao

**Affiliations:** 1 School of Mechanical Engineering, Sichuan University, Chengdu, 610065 China; 2 Engineering Research Center of Combustion and Cooling for Aerospace Power, Ministry of Education, Sichuan University, Chengdu, 610065 China

**Keywords:** Phase transitions and critical phenomena, Thermodynamics, Fluid dynamics

## Abstract

Although many theoretical and experimental studies on convective heat transfer exist, the consistent analytical expression of advection heat flux vector in convection as well as its reference temperature in the thermal driving force remains unclear. Here we show theoretically and experimentally the unifying formulae for three-dimensional (3D) heat flux vector of forced and natural convections for compressible laminar flows based on the first law of thermodynamics. It is indicated for a single-phase compressible fluid that advection is no other than heat transfer owing to mass flow in the forms of enthalpy and mechanical energy by gross fluid movement, driven by the temperature difference between the fluid temperature and the potential temperature associated with the relevant adiabatic work done. A simple formula for the total convective heat flux vector of natural convection is also suggested and reformulated in terms of logarithmic density difference as the thermal driving force. The theoretical calculations agree well with the laminar flow experiment results. Our discovery of advection heat transfer for compressible flows caused by the temperature differential in which the potential temperature is regarded as the unifying reference temperature represents a previously unknown thermal driving mechanism. This work would bring fundamental insights into the physical mechanism of convective heat transfer, and opens up new avenue for the design, calculation and thermal management of the 3D convection heat flux problems using the novel thermal driving force for compressible laminar and turbulent flows.

## Introduction

Convection (also called advection) heat transfer refers to the transport of thermal energy from one point to another by a macroscopic fluid motion given by the fluid velocity vector, resulting from a spatial variation in temperature^1^. Thermal convection is universal phenomena in nature, and serves important purposes in a wide variety of energy transport systems. For instance, it accomplishes the warming of the atmosphere^2^, the mixing of the oceans^3^ and the heating of geothermal system^4^, reveals the driving mechanism of mantle convection^5–7^, predicts meteorology^8^, augments the bioheat transfer of blood vessels^9,10^ and heat transports of fuel cells^11^, and enhances the transpiration cooling of turbine blades^12^, rocket engines^13^ and hypersonic vehicles^14^. A basic law of heat flux underlies the design, calculation and optimization of any convection heat transfer process, and it should render the unambiguous property relationship between a heat flux vector and its thermal driving force^1,15–19^. The heat flux vector is defined as the thermal energy flow per unit time and per unit area^1^. Convective heat transfer always focuses on the convective heat flux vector **q,** including its magnitude, direction, and spatial and temporal variations in a fluid stream^1^. In this paper, we will examine **q** within a fluid stream without directly considering the heat transfer between a fluid and its adjacent solid surface. The total convection heat flux vector **q** through the flow field is a superposition of two heat transfer modes: advection (**q**_*u*_) due to gross fluid movement and conduction (**q**_*k*_) due to random molecular motion^15–17^. Hereafter *convection* refers to this cumulative transport and *advection* refers to transport due to bulk fluid motion. Although there are many analytical^1,8,9,15,16,21–31^, numerical^32^ and experimental^33,34^ studies on convective heat transfer, the basic properties of heat transport including the heat flux vector for compressible flows, remain unclear. It is widely accepted that the conduction heat flux vector **q**_*k*_ of **q** is governed by Fourier’s law of heat conduction^20^. However, a unifying theoretical expression for the advection heat flux vector **q**_*u*_ has not been widely agreed until now. Hence, **q**_*u*_ should be placed as the central focus of convective heat transfer. In some literature, the advection heat flux is expressed as **q**_*u*_ = *ρ***U***e*^1^ (*e* refers to specific internal energy, J/kg; *ρ* is fluid density, kg/m^3^, and **U** is fluid velocity vector, m/s.) or **q**_*u*_ = *ρ***U***e*^0^ [^15,21–25^]. (*e*^0^ is denoted as the combination of both *e* and specific mechanical energy *e*_*m*_, J/kg.) In other references, it is also presented by **q**_*u*_ = *ρc*_*p*_**U***T*^16,26^ (*c*_*p*_ represents specific heat capacity at constant pressure, J/(kg•K); *T* is temperature of fluid, K.) or **q**_*u*_ = *ρc*_*p*_**U**(*T* − *T*_*ref*_)^8,9,29^. (*T*_*ref*_ is reference temperature, K.) Furthermore, the concept of net energy flow^16,27,28^ and field synergy principle^30,31^ are also proposed on the basis of the equation of energy conservation, respectively. It should be pointed out that no any thermal driving force is revealed in the formulations of *ρ***U***e*, *ρ***U***e*^0^ and *ρc*_*p*_**U***T*, where both of *e* and *e*^0^ are not easy to determine since the properties of a flowing fluid may be varying with time and position. Although the temperature difference *T* − *T*_*ref*_ is considered to be the thermal driving potential for *ρc*_*p*_**U**(*T*–*T*_*ref*_), the physical definition and selection of *T*_*ref*_ are actually equivocal^27–29^. Above all, to the best of the author’s knowledge, no unified analytical expression for the advection heat flux vector has been so far proposed for compressible flows.

Here we theoretically and experimentally show the unifying formulae of three-dimensional (3D) heat flux vector in convection heat transfer within a compressible single-phase fluid stream, according to the first law of thermodynamics for open systems^1,23,35,36^. It is indicated that heat advection is the energy transport in which both of enthalpy and mechanical energy are transferred by the mass flow due to bulk fluid motion, driven by the temperature difference between fluid temperature and the potential temperature associated with the relevant adiabatic work done. The reference temperature in the expression of advection heat flux vector is clarified and unified as the potential temperature. It is further shown that the total convective heat flux vector of natural convection can be reformulated in terms of logarithmic density difference as the thermal driving force. The present convection theory is partially validated by carrying out the steady heat transfer experiment for an incompressible laminar flow inside the circular tube. Our result reveals a novel thermal driving mechanism of advection heat transfer through the temperature differential in which the potential temperature is regarded as the unifying reference temperature for compressible flows. It also opens up new opportunities for studying the design and thermal management of 3D convection heat flux problems using the suggested thermal driving force for compressible laminar and turbulent flows.

## Theory

### Heat flux vector in compressible flows

**Figure 1:**
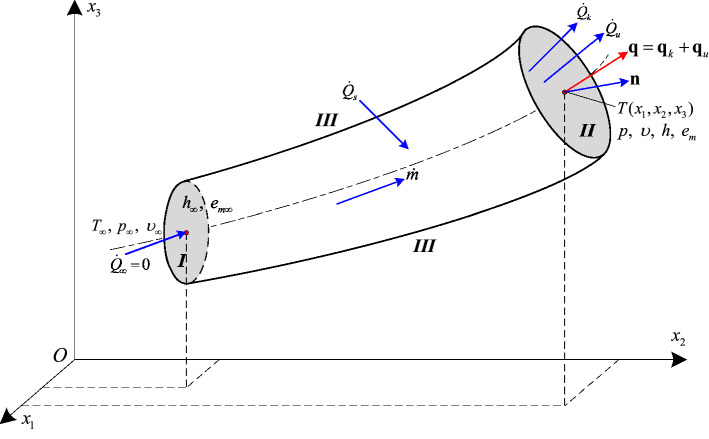
Sketch diagram of convection energy transfer. Control volume for steady compressible flow in a tube showing an advective heat transfer rate in a fluid-flow field in Cartesian coordinate system.

In order to obtain the unifying formulae for heat flux vector in compressible flows, the theory for convective heat transfer will be proposed and the concept of potential temperature will be introduced and developed. Although heat transfer is a result of thermal nonequilibrium in a medium or between media^1^, a local equilibrium state can still be used as a reasonable approximation^24,25,35^. A viscous dissipation is negligible, and no internal heat source (e.g. radiation, chemical reactions, Joule’s heat) is generated within the single-phase isotropic Newtonian fluid^1^. A steady compressible flow in a tube is considered, as shown in Fig. 1. The control volume is completely enclosed by the tube inlet surface *I*, some arbitrary inner surface *II* within a fluid stream along the tube at which convection heat transfer occurs, and the stiff wall surface *III* between *I* and *II*.

Fluid moves at a constant mass flow rate $\dot m$, and advection due to bulk fluid motion and conduction due to thermal diffusion relative to macroscopic fluid movement occur at surface *II*^24^. No shaft work is done by the fluid^15,23^ and only flow work (*pυ*) is performed to push fluid through the control surfaces *I* and *II*. Hence the significant convective heat transfer effect will mainly result from the heat advection at surface *II* associated with the changes of enthalpy (combination of internal energy and flow work) and mechanical energy (including kinetic and potential energies), as well as heat conduction in the axial direction. No heat transfer appears at the tube inlet (the heat transfer rate at the inlet ${\dot Q_\infty }$ is always assumed to be zero), and *h*_∞_ and *e*_*m*∞_ represent the specific enthalpy and specific mechanical energy at the inlet surface, respectively, and *T*_∞_, *p*_∞_ and *υ*_∞_ denote the inlet temperature, pressure and specific volume, respectively. All the parameters are presumed to be constant within the inlet surface. Likewise, *h*, *e*_*m*_, *T*, *p* and *υ* are respectively the specific enthalpy, specific mechanical energy, temperature, pressure and specific volume at surface *II*.

An energy balance through the control surfaces may be employed to determine how the heat transfer rate due to heat advection is related to the thermal driving potentials (Fig. 1):

1 $${\dot Q_s} + {\dot Q_\infty } = {\dot Q_k} + {\dot Q_u}$$

where ${\dot Q_s}$ is the heat transfer rate across the stiff wall surface (only by conduction), ${\dot Q_\infty }$ is the heat transfer rate through the tube inlet (${\dot Q_\infty } = 0$), and ${\dot Q_k}$ and ${\dot Q_u}$ are the heat transfer rate by conduction and advection through the flow section *II*, respectively.

By making the local thermodynamics equilibrium assumption^24,25,35,37^ for simple compressible substances and following the first law of thermodynamics for open systems^1,23,36,38^, as rendered in Fig. 1, one obtains

2 $${\dot Q_s} + {\dot Q_\infty } = {\dot Q_k} + \dot m\left[ {(h + {e_m}) - ({h_\infty } + {e_{m\infty }})} \right]$$

Comparing Eqs. (1) and (2), the heat transfer rate by heat advection due to bulk fluid motion ${\dot Q_u}$ can be determined as

3a $${\dot Q_u} = \dot m\left[ {(h - {h_\infty }) + ({e_m} - {e_{m\infty }})} \right]$$

Recasting the right-hand side of Eq. (3a) into the integral form between the surface *II* and the inlet surface *I*, it gives

3b $${\dot Q_u} = \dot m\int_I^{II} {{\text{(d}}h + {\text{d}}{e_m})}$$

Following the assumption of quasi-equilibrium process^24,25,35^ without shaft work and viscous dissipation, and taking the Bernoulli equation into account^25,39^, we have

4 $${\text{d}}{e_m} = - \upsilon {\text{d}}p$$

Inserting Eq. (4) into Eq. (3b) yields

5 $${\dot Q_u} = \dot m\int_I^{II} {{\text{(d}}h - \upsilon {\text{d}}p)}$$

By using the Gibbs equations and the Maxwell relation^1,36^, one obtains

6 $$\left\{ \begin{gathered} {\text{d}}p = {\left. {\frac{\partial p}{{\partial T}}} \right|_\upsilon }{\text{d}}T + {\left. {\frac{\partial p}{{\partial \upsilon }}} \right|_T}{\text{d}}\upsilon = \frac{\beta }{\kappa }{\text{d}}T - \frac{1}{\upsilon \kappa }{\text{d}}\upsilon \, \hfill \\ {\text{d}}\upsilon = {\left. {\frac{\partial \upsilon }{{\partial T}}} \right|_p}{\text{d}}T + {\left. {\frac{\partial \upsilon }{{\partial p}}} \right|_T}{\text{d}}p = \upsilon \beta {\text{d}}T - \upsilon \kappa {\text{d}}p \, \hfill \\ {\text{d}}T = {\left. {\frac{\partial T}{{\partial \upsilon }}} \right|_p}{\text{d}}\upsilon + {\left. {\frac{\partial T}{{\partial p}}} \right|_\upsilon }{\text{d}}p = \frac{1}{\upsilon \beta }{\text{d}}\upsilon + \frac{\kappa }{\beta }{\text{d}}p \hfill \\ \end{gathered} \right.$$

where *β* is volumetric coefficient of thermal expansion (K^-1^), *κ* is isothermal compressibility (Pa^−1^).

Note that the change of specific enthalpy *h* (J/kg) can be given in terms of two independent properties *T* and *p* or *T* and *υ*

7 $${\text{d}}h = {\left. {\frac{\partial h}{{\partial T}}} \right|_p}{\text{d}}T + {\left. {\frac{\partial h}{{\partial p}}} \right|_T}{\text{d}}p,{\text{ d}}h = {\left. {\frac{\partial h}{{\partial T}}} \right|_\upsilon }{\text{d}}T + {\left. {\frac{\partial h}{{\partial \upsilon }}} \right|_T}{\text{d}}\upsilon$$

According to the Bridgman’s relations^36^, these first partial derivatives become in the form

8 $${\left. {\frac{\partial h}{{\partial T}}} \right|_p} = {c_p}, \, {\left. {\frac{\partial h}{{\partial p}}} \right|_T} = (1 - \beta T)\upsilon , \, {\left. {\frac{\partial h}{{\partial T}}} \right|_\upsilon } = {c_\upsilon } + \frac{\beta \upsilon }{\kappa }{\left. {, \, \frac{\partial h}{{\partial \upsilon }}} \right|_T} = \frac{\beta T - 1}{\kappa }$$

where *c*_*p*_ (*c*_*υ*_) is specific heat capacity at constant pressure (volume) [J/(kg•K)]. Substituting Eq. (8) into Eq. (7) and combing Eq. (6), the integrand in Eq. (5) can be recast into

9 $${\text{d}}h - \upsilon {\text{d}}p = {c_p}{\text{d}}T - \beta T\upsilon {\text{d}}p,{\text{ d}}h - \upsilon {\text{d}}p = {c_\upsilon }{\text{d}}T + {{\beta T} \mathord{\left/ {\vphantom {{\beta T} \kappa }} \right. \kern-\nulldelimiterspace} \kappa }{\text{d}}\upsilon$$

Integrating Eq. (9) from the *T*_∞_ and *p*_∞_ (*υ*_∞_) state at the inlet surface *I* to the *T* and *p* (*υ*) state at some arbitrary inner surface *II* within a fluid stream, and considering the definition of heat transfer rate $\dot Q$, Eq. (5) can be rewritten as

10 $${\dot Q_u} = \int_A {({{\mathbf{q}}_u} \cdot {\mathbf{n}}){\text{d}}A} = \dot m\left( {\int_{T_\infty }^T {{c_p}{\text{d}}T^{\prime} - \int_{p_\infty }^p {\beta T\upsilon {\text{d}}p^{\prime}} } } \right) = \dot m\left( {\int_{T_\infty }^T {{c_\upsilon }{\text{d}}T^{\prime} + \int_{\upsilon_\infty }^\upsilon {{{\beta T} \mathord{\left/ {\vphantom {{\beta T} \kappa }} \right. \kern-\nulldelimiterspace} \kappa }{\text{d}}\upsilon ^{\prime}} } } \right)$$

where **q**_*u*_ is the advection heat flux (W/m^2^) through the surface *II*; *A* is the cross sectional area of surface *II*; **n** is the unit vector pointing outward, normal to the surface *A*, as shown in Fig. 1; $\dot m$ is mass flow rate (kg/s), by definition^35,38^, $\dot m = \int_A {\rho {\mathbf{U}} \cdot {\mathbf{n}}} {\text{d}}A$, where *ρ* is fluid density (kg/m^3^), and ${\mathbf{U}} = \left\{ {{u_1},{u_2},{u_3}} \right\}$ designates the velocity vector of a flowing fluid. The subscript ∞ refers to constant physical quantity at the inlet for internal flows, and to physical quantity at free-stream condition for external flows.

The sufficiently small differential mass is considered to possess uniform properties^35,40^, therefore, when inserting the definition of $\dot m = \int_A {\rho {\mathbf{U}} \cdot {\mathbf{n}}} {\text{d}}A$ into Eq. (10), the bracket terms on the right-hand side of Eq. (10) may be directly combined with the integrand of the definition of $\dot m$; when ∆*A* → 0, dropping the signs for integrals on both sides, it gives

11 $$\boxed{\left\{ \begin{gathered} {{\mathbf{q}}_u} = \rho {\mathbf{U}}\left( {\int_{T_\infty }^T {{c_p}{\text{d}}T^{\prime}} - \int_{p_\infty }^p {{{\beta T} \mathord{\left/ {\vphantom {{\beta T} \rho }} \right. \kern-\nulldelimiterspace} \rho }{\text{d}}p^{\prime}} } \right) \hfill \\ {{\mathbf{q}}_u} = \rho {\mathbf{U}}\left( {\int_{T_\infty }^T {{c_\upsilon }{\text{d}}T^{\prime}} + \int_{\upsilon_\infty }^\upsilon {{{\beta T} \mathord{\left/ {\vphantom {{\beta T} \kappa }} \right. \kern-\nulldelimiterspace} \kappa }{\text{d}}\upsilon ^{\prime}} } \right) \hfill \\ \end{gathered} \right.}$$

The above equations represent the general unified theoretical formulae of advective heat flux within a fluid stream for compressible flows.

Furthermore, the conduction heat flux **q**_*k*_, can be given by Fourier’s law^20^

12 $${{\mathbf{q}}_k} = - k\nabla T$$

where *k* is thermal conductivity [W/(m•K)]. Therefore, for a single-phase, compressible, isotropic Newtonian fluid, the total convection heat flux vector **q**(*x*_1_, *x*_2_, *x*_3_) = {*q*_1_, *q*_2_, *q*_3_} at any section within a flow field, as shown in Fig. 1 in Cartesian system, is the resultant of advective heat flux **q**_*u*_ and conductive heat flux **q**_*k*_^1,15–17,21–24,41^

13 $$\boxed{\left\{ \begin{gathered} {\mathbf{q}} = {{\mathbf{q}}_u} + {{\mathbf{q}}_k} = \rho {\mathbf{U}}\left( {\int_{T_\infty }^T {{c_p}{\text{d}}T^{\prime}} - \int_{p_\infty }^p {{{\beta T} \mathord{\left/ {\vphantom {{\beta T} \rho }} \right. \kern-\nulldelimiterspace} \rho }{\text{d}}p^{\prime}} } \right) - k\nabla T \hfill \\ {\mathbf{q}} = {{\mathbf{q}}_u} + {{\mathbf{q}}_k} = \rho {\mathbf{U}}\left( {\int_{T_\infty }^T {{c_\upsilon }{\text{d}}T^{\prime}} + \int_{\upsilon_\infty }^\upsilon {{{\beta T} \mathord{\left/ {\vphantom {{\beta T} \kappa }} \right. \kern-\nulldelimiterspace} \kappa }{\text{d}}\upsilon ^{\prime}} } \right) - k\nabla T \hfill \\ \end{gathered} \right.}$$

If the variations of *c*_*p*_ and *c*_*υ*_ with *T* and *p* (or *υ*) are relatively small, Eq. (11) can be therefore recast into

14 $$\left\{ \begin{gathered} {{\mathbf{q}}_u} = \rho {c_p}{\mathbf{U}}\left( {T - {T_\infty } - \int_{p_\infty }^p {\frac{\beta T}{{\rho {c_p}}}{\text{d}}p^{\prime}} } \right) \hfill \\ {{\mathbf{q}}_u} = \rho {c_\upsilon }{\mathbf{U}}\left( {T - {T_\infty } - \int_{\upsilon_\infty }^\upsilon {\frac{ - \beta T}{{\kappa {c_\upsilon }}}{\text{d}}\upsilon ^{\prime}} } \right) \hfill \\ \end{gathered} \right.$$

The temperature change resulted from the dynamic pressure (or density) variation in a compressible flow is one of the most important features for convection heat transfer. In particular, it appears useful to compare the temperature differences due to the change of enthalpy flow with those caused by the variation of pressure or density. Now let us examine the integral term in the bracket in Eq. (14) given in terms of the independent variable *p* or *υ*. For simplicity, it is permissible to assume that the variation process of pressure is adiabatic and reversible, because the small conductivity for a flowing medium and the high rate of change in the thermodynamic properties of state will, in general, prevent any appreciable heat transfer between the element of fluid with its surroundings^25^. Therefore, this integration term, under some circumstances, may be only associated with the *temperature difference in the adiabatic process*, resulting from the large change of pressure or specific volume for non-phase-change flows. Although an adiabatic temperature difference is produced, no heat is transferred across the element of fluid, so this temperature difference must be deducted from the total temperature difference (Δ*T* = *T* − *T*_∞_), as indicated in Eq. (14).

If the potential temperature function *T*_*ad*_ (*r*) is defined to be the temperature that an element of fluid would have if it were moved adiabatically from *r* = 0, the inlet position in a flow field, to *r*, some arbitrary position, then^7^

15 $${T_{ad}}(0) = {T_\infty }$$

and according to Eq. (14)

16a $${\text{d}}T_{ad,p} = \frac{{\beta T_{ad,p}}}{{\rho {c_p}}}{\text{d}}p$$

16b $${\text{d}}T_{ad,\upsilon } = - \frac{{\beta T_{ad,\upsilon }}}{{\kappa {c_\upsilon }}}{\text{d}}\upsilon$$

To explain the physical meaning of potential temperature, two particular applications will be considered and calculated. (i) *The adiabatic dry air with vertical motion in the atmosphere.* The dry air is treated as an ideal gas, then $\beta {T_{ad}}_{,p} = 1$. Applying the ideal gas equation of state to Eq. (16a), then employing some algebraic manipulations yields

17 $${\text{dln}}{T_{ad,p}} = {{[(\gamma - 1)} \mathord{\left/ {\vphantom {{[(\gamma - 1)} \gamma }} \right. \kern-\nulldelimiterspace} \gamma }{\text{]dln}}p$$

where *γ* is the specific heat ratio. Integrating both sides of Eq. (17) from the fluid inlet to some arbitrary position within a flowing field, and considering Eq. (15) gives the potential temperature (*T*_*ad*, *p*_) and related adiabatic temperature difference (Δ*T*_*ad*, *p*_ = *T*_*ad*, *p*_ − *T*_∞_), respectively

18a $${T_{ad,p}} = {T_\infty }{\left( {{p \mathord{\left/ {\vphantom {p {p_\infty }}} \right. \kern-\nulldelimiterspace} {p_\infty }}} \right)^{\frac{\gamma - 1}{\gamma }}}, \, \Delta {T_{ad,p}} = {T_\infty }{\left( {{p \mathord{\left/ {\vphantom {p {p_\infty }}} \right. \kern-\nulldelimiterspace} {p_\infty }}} \right)^{\frac{\gamma - 1}{\gamma }}} - {T_\infty }$$

Notice that the above results become the identical adiabatic process equations for an ideal gas^35,38,40^.

Likewise, if the dry air is considered as a real gas, integrating Eq. (16b) with the boundary condition (15) and employing some algebraic manipulations yields

18b $${T_{ad,\upsilon }} = {T_\infty }{e^{ - \frac{\beta }{{\kappa {c_\upsilon }}}(\upsilon - {\upsilon_\infty })}}, \, \Delta {T_{ad,\upsilon }} = {T_\infty }{e^{ - \frac{\beta }{{\kappa {c_\upsilon }}}(\upsilon - {\upsilon_\infty })}} - {T_\infty }$$

provided *β*/(*κc*_*υ*_) is not a function of *υ*.

(ii) *The adiabatic convection fluid within the mantle.* The adiabatic convection condition is satisfied within the mantle because the thermal conductivity of rocks is relatively small^6,7^. Within the earth, we have^7^

19 $${\text{d}}p = - \rho g{\text{d}}r$$

where *g* is the acceleration of gravity. Substituting Eq. (19) into Eq. (16a) so that

20 $${\text{dln}}{T_{ad,p}} = - [\beta g/{c_p}]{\text{d}}r$$

If the initial distance from the center of the earth is *r*_0_ within a fluid stream, considering the boundary condition ${T_{ad}}(r = {r_0}) = {T_\infty }$, and integrating Eq. (20) gives

21 $${T_{ad,p}} = {T_\infty }{e^{ - {{\beta g(r - {r_0})} \mathord{\left/ {\vphantom {{\beta g(r - {r_0})} {c_p}}} \right. \kern-\nulldelimiterspace} {c_p}}}}, \, \Delta {T_{ad,p}} = {T_\infty }{e^{ - {{\beta g(r - {r_0})} \mathord{\left/ {\vphantom {{\beta g(r - {r_0})} {c_p}}} \right. \kern-\nulldelimiterspace} {c_p}}}} - {T_\infty }$$

provided *βg*/*c*_*p*_ is not a function of *r*.

Therefore, the advection heat flux in Eq. (14) for compressible flows can be rewritten, in the form of thermal driving force, namely, the effective temperature difference (Δ*T* − Δ*T*_*ad*_), as follows

22 $${{\mathbf{q}}_u} = \rho {c_p}{\mathbf{U}}(T - {T_{ad,p}}), \, {{\mathbf{q}}_u} = \rho {c_\upsilon }{\mathbf{U}}(T - {T_{ad,\upsilon }})$$

Accordingly, the total convective heat flux in Eq. (13) becomes

23 $$\boxed{\left\{ \begin{gathered} {\mathbf{q}} = {{\mathbf{q}}_u} + {{\mathbf{q}}_k} = \rho {c_p}{\mathbf{U}}(T - {T_{ad,p}}) - k\nabla T \hfill \\ {\mathbf{q}} = {{\mathbf{q}}_u} + {{\mathbf{q}}_k} = \rho {c_\upsilon }{\mathbf{U}}(T - {T_{ad,\upsilon }}) - k\nabla T \hfill \\ \end{gathered} \right.}$$

For conduction, heat flows in the direction of decreasing temperature. When a moving fluid is present for advection, however, heat flows along the same or opposite direction as the fluid velocity vector **U**, as shown in Eq. (23). Obviously, advection can enhance or weaken conduction, depending on the direction of flowing velocity. The above convection heat flux formulae for compressible flows also have potentials to be applied to the actively cooled structures such as rocket engines and hypersonic vehicles under high aerodynamic thermal loads^12–14^.

### Heat flux vector in incompressible flows

In particular, when flow velocity is not higher than one quarter of the speed of sound, the variation of pressure (or specific volume) can be neglected. Then the fluid can be treated as an incompressible medium^21,25^, namely, *β* = 0. Following Eqs. (15), (16a) and (16b), the potential temperature function *T*_*ad*_ reduces to

24 $${T_{ad}} = {T_\infty }$$

Hence the adiabatic temperature difference vanishes, i.e., $\Delta {T_{ad}}_{,p} = 0$, the Eqs. (11), (14), (22) and (23) degenerate into

25 $${{\mathbf{q}}_u} = \rho {\mathbf{U}}\int_{T_\infty }^T {{c_p}{\text{d}}T^{\prime}} {\text{ or }}{{\mathbf{q}}_u} = \rho {c_p}{\mathbf{U}}(T - {T_\infty })$$

26a $${\mathbf{q}} = {{\mathbf{q}}_u} + {{\mathbf{q}}_k} = \rho {c_p}{\mathbf{U}}(T - {T_\infty }) - k\nabla T$$

where the difference of *c*_*p*_ and *c*_*υ*_ is thought to be negligible for the incompressible fluids. It is worth noting that Eq. (26a) is identical to the 3D heat flux vector proposed by reference^29^ for incompressible flows and also reduces to “*net energy flows*” of two-dimensional (2D) flows by Bejan et al^27,28^. The convective heat flux vector **q** in Eq. (26a) can be recast in terms of its vectorial components as

26b $${q_i} = - \rho {c_p}a\frac{\partial T}{{\partial {x_i}}} + \rho {c_p}{u_i}(T - {T_\infty }) = - k\frac{\partial T}{{\partial {x_i}}}\left[ {1 + \frac{{{u_i}/a}}{{{{\partial T/\partial {x_i}} \mathord{\left/ {\vphantom {{\partial T/\partial {x_i}} {({T_\infty } - T)}}} \right. \kern-\nulldelimiterspace} {({T_\infty } - T)}}}}} \right]$$

where *i* = 1,2,3, $a = {k \mathord{\left/ {\vphantom {k {(\rho {c_p}}}} \right. \kern-\nulldelimiterspace} {(\rho {c_p}}})$ is molecular thermal diffusivity (m^2^/s). It is indicated from Eq. (26b) for incompressible laminar flows that at any instant, in addition to the random motion of molecules retained in the aggregate (i.e., conduction, thermal diffusivity *a* is analogous to “*relative velocity*” of convection) , a large number of molecules also move collectively by some macroscopic velocity in the *x*_*i*_ direction (i.e., advection, *u*_*i*_ is analogous to “*velocity of entrainment*” of convection) and carry all the energy of aggregate at the temperature *T*_∞_ (including *internal energy* and *flow work*). When this aggregate with temperature *T*_∞_ remains moving forward and meets another aggregate with temperature *T* at some position in the flow field, the two aggregates will collide and mix each other within the same flowing surface, and exchange their respective enthalpies much more rapidly than would take place by conduction alone in the same medium if restrained from moving. The amount of heat transferred per unit time per unit area in this direction due to advection is proportional to the temperature difference *T*–*T*_∞_ and *ρu*_*i*_, which can be well explained by kinetic molecular theory^24,42,43^. Owing to bulk fluid motion, the isothermal surfaces are so extended that their areas are greatly increased while the distances between them are greatly diminished or even zero, hence the energy exchange between the new and old fluid bulks almost occurs at the same position where the collision frequency and motion velocity of fluid particles are so high that energy can be transferred at a very high rate. As a result, energy is transferred from the hot to the cold part. As seen in Eq. (26b), the rate of advection heat transfer in *x*_*i*_ direction will have $\left| {\frac{{{u_i}/a}}{{{{\partial T/\partial {x_i}} \mathord{\left/ {\vphantom {{\partial T/\partial {x_i}} {({T_\infty } - T)}}} \right. \kern-\nulldelimiterspace} {({T_\infty } - T)}}}}} \right|$ times as much as conduction, and this value appears much larger than 1. Therefore, the present theory confirms the viewpoints of convective heat transfer by White^41^ and Maxwell^44^ quantitatively.

### Fourier’s law of conduction and Newton’s law of cooling

The two limit cases are of special interest here. (a) If the fluid flow subsides (${\mathbf{U}} = 0$), **q**_*u*_ in **q** turns into zero, and Eqs. (13), (26a) and (26b) become identical to Fourier’s law of heat conduction^20^. (b) Considering the convective heat transfer between a solid surface and the adjacent fluid moving over the surface for external flows. If the solid is small in size and its temperature has not so large that their energy is not sufficient to change the surrounding temperature of fluid^45^. When the thermal diffusivity of the incompressible fluid tends infinity (i.e., *a* → ∞, hence no temperature gradient and no conduction inside the flow field^46^), only the advective terms are retained in Eq. (26b) and the fluid temperature is considered to be the free-stream temperature *T*_∞_ anywhere except the body surface. Accordingly, no boundary layer is developed within the adjacent fluid stream, hence the velocity of the flowing fluid anywhere is considered to be uniform and constant, i.e., the free-stream velocity *u*_∞_^45,46^. Apparently, this problem reduces to the Newton’s cooling problem whose heat transfer only depends on heat advection^46,47^. Therefore, the energy transfer at the interface between the body surface and the adjacent fluid, only due to heat advection, can be treated as a lumped-parameter model. According to Eq. (26b), we have the surface advective heat flux ${q_s} = \rho {c_p}{u_\infty }({T_s}-{T_\infty })$, where *T*_*s*_ is the body’s uniform temperature at wall surfaces. From the Newton’s original cooling formula^46,47^, one obtains ${q_s} = {h_{Newton}}({T_s}-{T_\infty })$, where ${h_{Newton}}$ is the cooling coefficient of Newton’s original rate equation ^46,47^. Equating the two yields

27 $${h_{Newton}} = \rho {c_p}{u_\infty }$$

It is observed that ${h_{Newton}}$ equals to the product of volumetric heat capacity and free-stream velocity. It is worth noting that the Newton’s cooling coefficient ${h_{Newton}}$, which is different from the convective heat transfer coefficient (historically proposed by Fourier^20,46^), remains constant and is only associated with the physical properties of fluid and *u*_∞_. This conclusion can be partially validated by the experiment by O’Sullivan^48^ in which the Newton’s cooling coefficient is indicated to be proportional to the free-stream velocity *u*_∞_.

### Heat flux vector and energy equation of conservation

Heat transfer is a result of temperature nonuniformity. This variation in temperature is governed by the energy equation of conservation (with a reformulation that places **q** as the central focus)^1^. It is assumed that the specific total energy *e*_*t*∞_ within the control volume *V* remains constant for steady flows (see Fig. 1), then the case of unsteady flows is further considered. When time elapses from *t* to *t* + ∆*t*, specific total energy varies from *e*_*t*∞_ to *e*_*t*_. Hence integrating Eq. (9) leads to the rate of change of total energy ${\dot E_{CV}}$ stored within the control volume^40,49,50^

28 $${\dot E_{CV}} = \frac{{\text{d}}}{{{\text{d}}t}}\int_V {\rho ({e_t} - {e_{t\infty }}{\text{)d}}V} = \frac{{\text{d}}}{{{\text{d}}t}}\int_V {\rho \left[ {(h - {h_\infty }) + ({e_m} - {e_{m\infty }})} \right]{\text{d}}V} = \frac{{\text{d}}}{{{\text{d}}t}}\int_V {\rho \left( {\int_{T_\infty }^T {{c_p}{\text{d}}T^{\prime} - \int_{p_\infty }^p {\beta T\upsilon {\text{d}}p^{\prime}} } } \right){\text{d}}V}$$

where *e*_*t*_ is the aforementioned specific total energy consisting of specific enthalpy *h* and mechanical energy *e*_*m*_^40^. When ∆*V* → 0, dropping the signs of integration on both sides of Eq. (28), it gives

29 $$\frac{{{\text{d(}}\rho \Delta {e_t})}}{{{\text{d}}t}} = \frac{{\text{d}}}{{{\text{d}}t}}\left[ {\rho \left( {\int_{T_\infty }^T {{c_p}{\text{d}}T^{\prime} - \int_{p_\infty }^p {\beta T\upsilon {\text{d}}p^{\prime}} } } \right)} \right]$$

Neglecting a viscous dissipation and internal heat source, considering continuity equation, *υ* = 1/*ρ* and Eq. (29), and inserting Eq. (13) into the elemental energy balance relationship $\frac{{{\text{d(}}\rho \Delta {e_t})}}{{{\text{d}}t}} + \nabla \cdot {\mathbf{q}} = 0$^1,21–23^ for unsteady heat transfer process (see Fig. 1) leads to the energy equation of conservation for compressible laminar flows identically^1,21,22,25^

30 $$\nabla \cdot (k\nabla T) = \rho {c_p}\frac{{{\text{D}}T}}{{{\text{D}}t}} - \beta T\frac{{{\text{D}}p}}{{{\text{D}}t}} = \rho {c_\upsilon }\frac{{{\text{D}}T}}{{{\text{D}}t}} - \frac{\beta T}{\kappa }\frac{{{\text{D}}\ln \rho }}{{{\text{D}}t}}$$

where D(∙)/D*t* is the substantial derivative in rectangular coordinates. It is also worth noting that substituting (26a) into ∇∙**q** = 0 for 2D steady incompressible flows, then integrating within the laminar boundary layer and employing some algebraic manipulations, one obtains the field synergy principle proposed by Guo et al^30^.

For incompressible fluid flows, the convection heat flux vector **q** in Eq. (26a) is also given in terms of its vectorial components (*q*_*r*_, *q*_*φ*_, *q*_*x*_) in the cylindrical coordinate system:

31 $${q_r} = \rho {c_p}{u_r}(T - {T_\infty }) - k\frac{\partial T}{{\partial r}}, \, {q_\varphi } = \rho {c_p}{u_\varphi }(T - {T_\infty }) - \frac{k}{r}\frac{\partial T}{{\partial \varphi }}, \, {q_x} = \rho {c_p}{u_x}(T - {T_\infty }) - k\frac{\partial T}{{\partial x}}$$

where *q*_*r*_, *q*_*φ*_, *q*_*x*_ are the heat flux components, and *u*_*r*_, *u*_*φ*_, *u*_*x*_ are the velocity components in radial, circumferential and axial directions, respectively.

### Heat flux vector for natural convection

Specifically, the heat flux vector of natural convection for a compressible flowing medium is considered. The principle of the local state is still valid for natural convecton^25,35^. Considering the relation *υ* ≡ 1/*ρ*, if the difference between pressure (or density) and its free-stream value is relatively small, the third equation of Eq. (6) can be simplified as

32 $$T - {T_\infty } = {{\left[ {\kappa (p - {p_\infty }) - \ln ({\rho \mathord{\left/ {\vphantom {\rho {\rho_\infty }}} \right. \kern-\nulldelimiterspace} {\rho_\infty }})} \right]} \mathord{\left/ {\vphantom {{\left[ {\kappa (p - {p_\infty }) - \ln ({\rho \mathord{\left/ {\vphantom {\rho {\rho_\infty }}} \right. \kern-\nulldelimiterspace} {\rho_\infty }})} \right]} \beta }} \right. \kern-\nulldelimiterspace} \beta }$$

The effect of pressure difference can be ignored in flows which are affected by gravitation^15,17,21,22,25,41^, Eq. (32) reduces

33a $$T - {T_\infty } = - {{\ln ({\rho \mathord{\left/ {\vphantom {\rho {\rho_\infty }}} \right. \kern-\nulldelimiterspace} {\rho_\infty }})} \mathord{\left/ {\vphantom {{\ln ({\rho \mathord{\left/ {\vphantom {\rho {\rho_\infty }}} \right. \kern-\nulldelimiterspace} {\rho_\infty }})} \beta }} \right. \kern-\nulldelimiterspace} \beta }{\text{ or }}{\rho \mathord{\left/ {\vphantom {\rho {\rho_\infty }}} \right. \kern-\nulldelimiterspace} {\rho_\infty }} = {e^{ - \beta (T - {T_\infty })}}$$

Expanding the right-hand side of Eq. (33a) into the power series and only retaining the linear term yields

33b $$1 - {\rho/ {\rho_\infty }} \approx - \ln ({\rho/ {\rho_\infty }})$$

By differentiating Eq. (33a) with respect to *x*_*i*_ (*i* = 1,2,3), we get

33c $$\frac{\partial T}{{\partial {x_i}}} = - \frac{1}{\beta }\frac{{\partial \ln ({\rho \mathord{\left/ {\vphantom {\rho {\rho_\infty }}} \right. \kern-\nulldelimiterspace} {\rho_\infty }})}}{{\partial {x_i}}}$$

Considering the relation *υ* ≡ 1/*ρ*, substituting Eq. (18b) into Eq. (22) then expanding the right-hand side into the power series and only retaining the linear part gives

34 $${{\mathbf{q}}_u} = \rho {c_\upsilon }{\mathbf{U}}(T - {T_\infty }) + \frac{{\beta {T_\infty }}}{\kappa }{\mathbf{U}}{{({\rho_\infty } - \rho )} \mathord{\left/ {\vphantom {{({\rho_\infty } - \rho )} {\rho_\infty }}} \right. \kern-\nulldelimiterspace} {\rho_\infty }}$$

The total convection heat flux accordingly becomes

35 $${\mathbf{q}} = {{\mathbf{q}}_u} + {{\mathbf{q}}_k} = \rho {c_\upsilon }{\mathbf{U}}(T - {T_\infty }) + \frac{{\beta {T_\infty }}}{\kappa }{\mathbf{U}}{{({\rho_\infty } - \rho )} \mathord{\left/ {\vphantom {{({\rho_\infty } - \rho )} {\rho_\infty }}} \right. \kern-\nulldelimiterspace} {\rho_\infty }} - k\nabla T$$

Generally, the flow velocity is relatively small for natural convection processes, thus the difference of temperature (or density) is not so large. Inserting Eqs. (33a)-(33c) into Eq. (35) and considering ${c_p} - {c_\upsilon } = {\beta^2}T\upsilon /\kappa$^1,36^, one arrives at the total heat flux component for natural convection

36 $$\boxed{{q_i} = \frac{{\rho {c_p}}}{\beta }\left[ {a\frac{{\partial \ln \rho /{\rho_\infty }}}{{\partial {x_i}}} - {u_i}\ln (\rho /{\rho_\infty })} \right]}$$

where *i* = 1,2,3, and *a* = *k*/(*ρc*_*p*_) is molecular thermal diffusivity (m^2^/s). It is worth noting that the above equation can be applied to calculate natural convective heat flux for laminar compressible flows with the variable properties, which makes it possible to be not limited to the Boussinesq approximation^1,15,17,21,22,25,41^. It is also shown from Eq. (36) that the *logarithmic difference of fluid density instead of temperature difference, can be regarded as the thermal driving potential of natural convection processes*. Inserting (36) into ∇•**q** = 0 and considering the continuity equation leads to the energy conservation equation for steady compressible flows

37 $${c_p}{\mathbf{U}} \cdot \nabla \rho = \nabla \cdot (k\nabla {\text{ln}}\rho )$$

Noting that the variable *T* in previous energy equation is substituted by the variable *ρ* here, thus the new energy equation may be easily solved numerically, in conjunction with the continuity equation and momentum equations (Navier–Stokes equations) due to the same variable *ρ* embedded in them.

## Experiment

### Experimental setup

**Figure 2:**
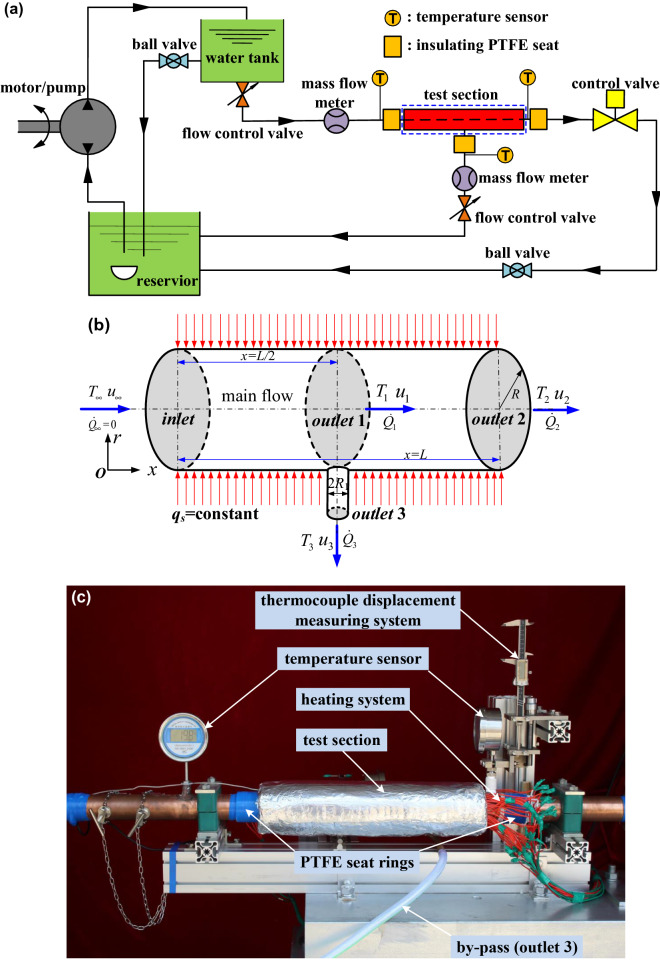

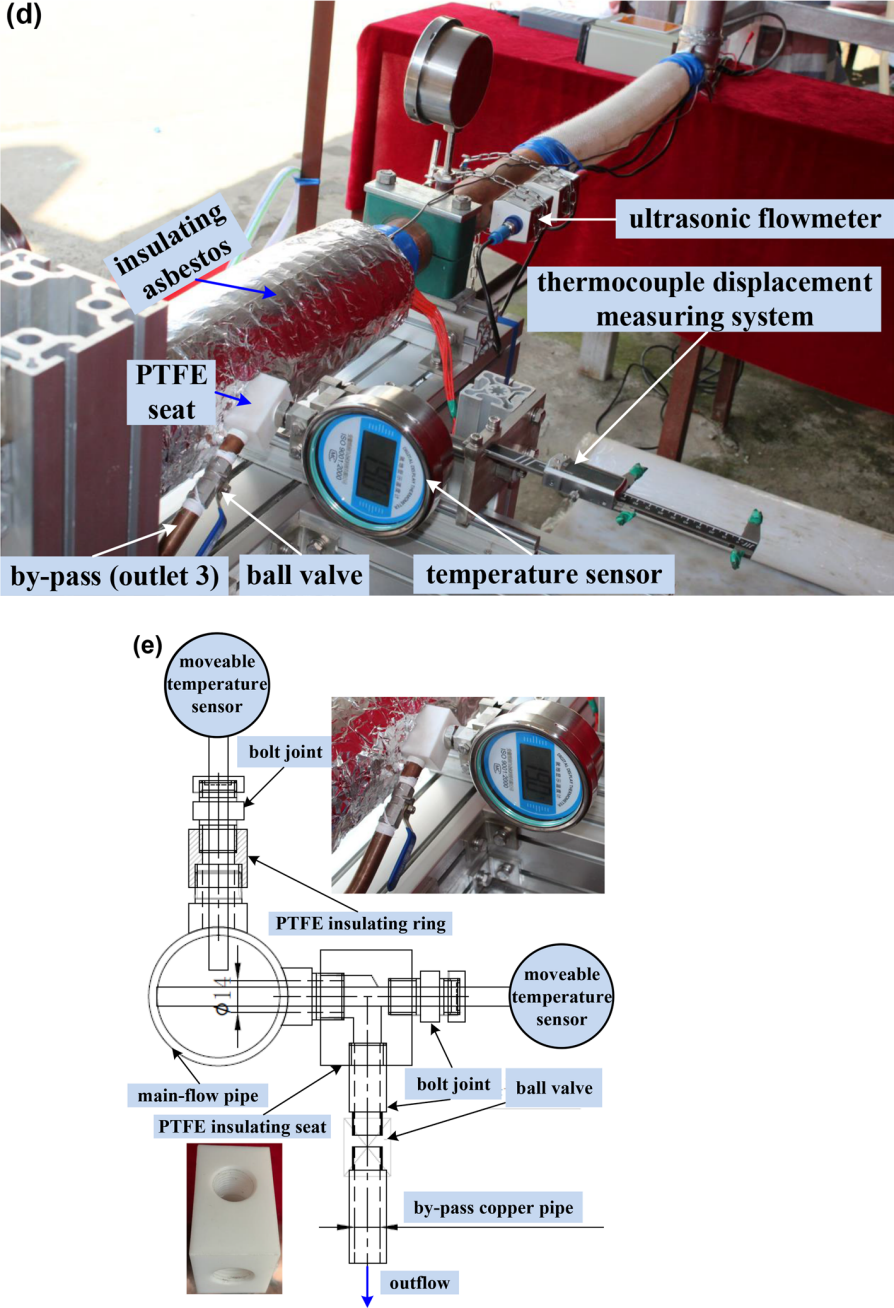
Experimental setup. (**a**) The layout of the laminar flow test rig. (**b**) Test section of the laminar flow geometry. (**c**) Front view of experimental setup and (**d**) Rear view of experimental setup. (**e**) Installation and connection of the by-pass copper tube at outlet 3.

In order to verify the above heat flux theory derived, a test facility is designed and constructed to investigate the steady convection heat transfer characteristic for an incompressible laminar flow in the circular tube with multiple outlets. The purpose of selecting a multiple-outlet test section is to clarify the concept of reference temperature (*T*_*ref*_), and to emphasize the importance of inlet temperature or free-stream temperature (*T*_∞_) for the convective heat flux formula proposed. The details of the test rig are shown in Fig. 2(a). The experimental setup consists of the water supply unit (motor/pump, water tank and water control unit), upstream and downstream tubes, test section, flow control valves, and temperature, velocity and rate of flow measuring systems. The test section, as shown in Fig. 2(b), consists the main pipe with single inlet and multiple outlets, and *T*_∞_ (*u*_∞_, ${\dot Q_\infty }$), *T*_1_ (*u*_1_, ${\dot Q_1}$), *T*_2_ (*u*_2_, ${\dot Q_2}$) and *T*_3_ (*u*_3_, ${\dot Q_3}$) are denoted as the mean temperatures (average flow velocities parallel to the inlet/outlet axis, rates of heat transfer) of water at the cross sections of inlet, outlet 1, 2 and 3, respectively. The experimental test element is made in a shape of the circular copper tube *R* = 26.5 ± 0.01 mm in inner radius and *L* = 460 ± 0.5 mm long, with one main-flow outlet and one by-pass outlet (inner radius *R*_1_ = 7 ± 0.01 mm) at the central part. As indicated in Figs. 2(c–d), there are 34 stainless steel heating rods to be installed on the pipe surface densely and uniformly, to provide the constant surface heat flux *q*_*s*_ with maximum heating power of 5765.8 W. In order to avoid heat loss, the external surfaces of these heating rods are thoroughly insulated with asbestine shroud to prevent radiation, and the inlet and all the outlets of pipe are bolted with the insulating elements of PEFE to prevent conduction. Both ends of the test section are also connected with the copper pipes with the same material and diameter as the main pipe. Figure 2(e) shows in detail how the by-pass tube is installed and thermally insulated. The steady laminar flow inside the experimental tube, whose Reynolds number is not more than 2500 for each test, is guaranteed by employing the special water control unit and mass flow controller to obtain the constant flow rate. The wall-normal temperature gradient [in the *r* direction in Fig. 2(b)] across the cross section of outlet 3 is determined by the difference of temperature values between two different points along a very tiny radial distance, measured by the moveable temperature sensor driven by the displacement measuring system, as shown in Figs. 2(d-e) in detail. The temperature sensor is with a margin of relative error of 2% and the hypersonic flowmeter measured flow rate and velocity with 1% as well as 6% for rate of heat transfer.

### Experimental validation

**Figure 3:**
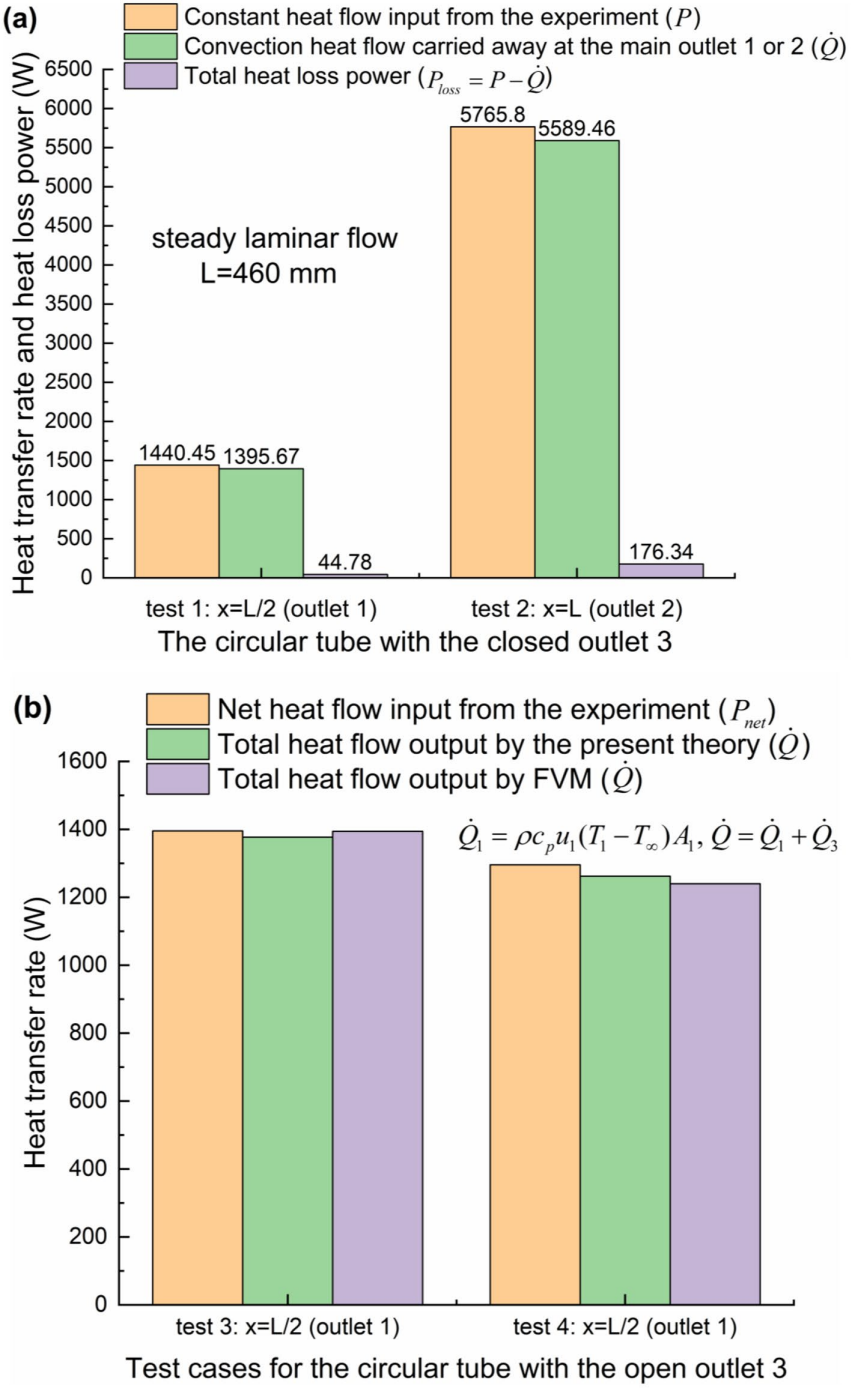

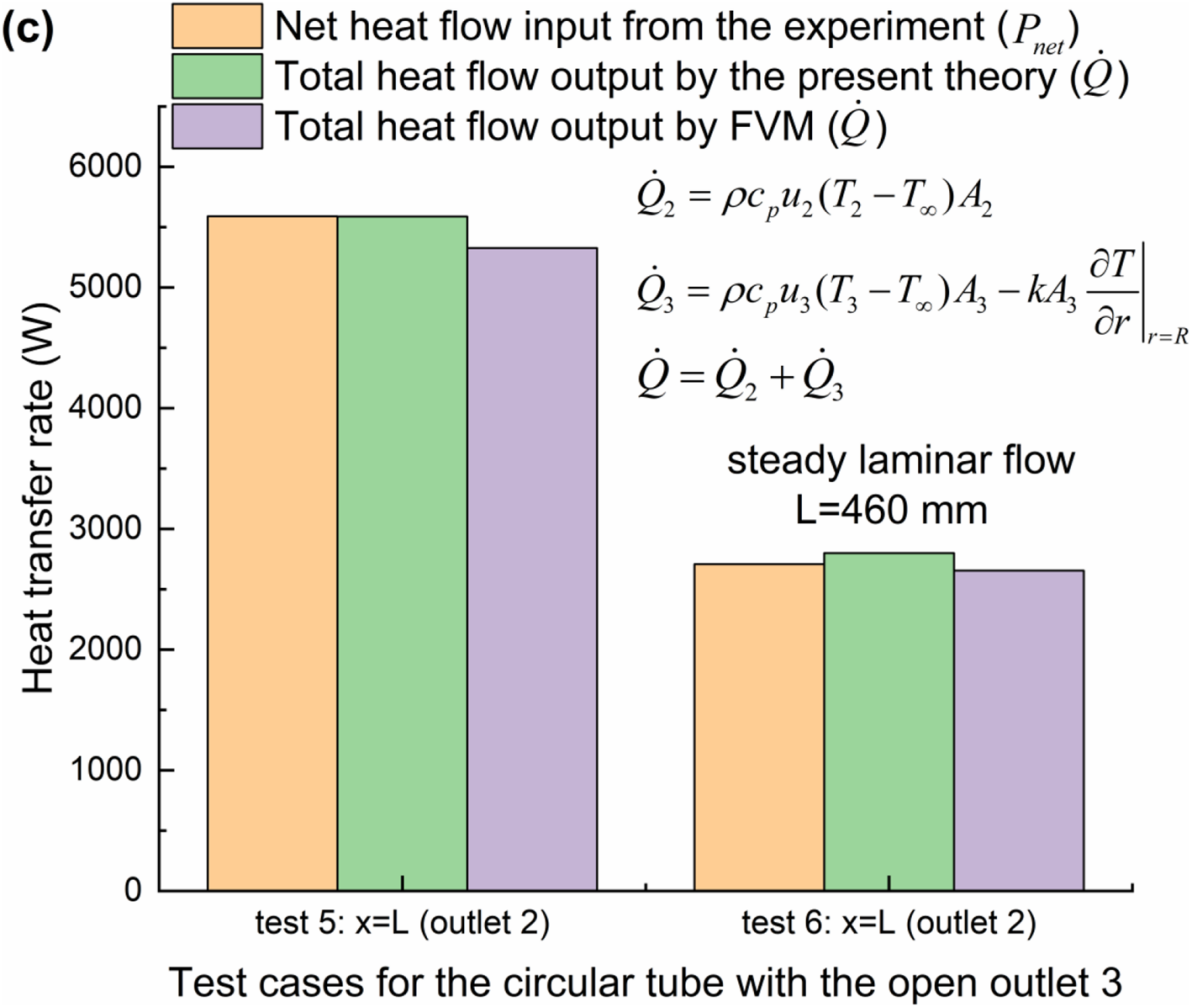
Verification of the present theory by the steady laminar flow experiments for convection heat transfer. (**a**) Determination of heat loss powers for the half-length and full-length SISO pipes. (**b**) Heat transfer rates obtained experimentally, theoretically and numerically for the SIMO pipes with the half-length and (**c**) full-length, where *A*_1_ (*A*_2_, *A*_3_) is the cross-sectional area of outlet 1 (2, 3).

During the implementation of steady convective laminar flow experiments, the outer diameter of by-pass tube in the test element is so small that the heat loss power can be approximated to be nearly equal when the same test conditions are applied except that the outlet 3 is open or closed [see Fig. 2(b)]. If the by-pass exit is closed, therefore, the heat loss powers *P*_*loss*_, can be firstly determined through the energy balance during the convective heat transfer process under the same condition of constant heating power *P* from the outer surface of circular pipe, considering steady laminar flows inside the left half part of pipe [i.e., from the inlet *x* = 0 to the half-length *x* = *L*/2] and inside the whole pipe (from the inlet *x* = 0 to the full-length *x* = *L*), as seen in Fig. 2(b), with the single-inlet & single-outlet (SISO) in test 1 and 2, respectively, as shown in Fig. 3(a). It is worth noting that the net outflow of heat transfer rate $\dot Q$ convected from the inlet to the outlet 1 or 2 is derived from Eq. (31) theoretically [neglecting the axial direction conduction^15^, as seen in Figs. 2(b) and 3(a)]. The heat transfer rate ${\dot Q_\infty } = 0$ is rendered because of *T* = *T*_∞_ at the inlet. Then the net inflow of heat transfer rate into the flowing fluid *P*_*net*_ is obtained for the single-inlet & multiple-outlet (SIMO) pipe by deducting the previously obtained heat loss power *P*_*loss*_ from the applied heating power. Therefore, *P*_*net*_ can be compared with the net outflow of heat transfer rate $\dot Q$, also determined by the convective heat flux formula (31), for the SIMO pipe with the half-length (*x* = *L*/2) in test 3–4 or the full-length (*x* = *L*) in test 5–6, as indicated in Figs. 3(b-c). The above comparison can be regarded as the experimental validation of the present theory. Similarly, the relatively small conduction in the streamwise direction (*x*) is neglected^15^. However, the wall-normal [parallel to the *r* direction in Fig. 2b] conduction of water across the section of outlet 3 needs to be treated cautiously. The magnitude of conduction heat flux of water is usually comparable to that of advection heat flux within the fluid phase inside the permeable porous media wall under some particular condition^32–34,51,52^. The total heat flow output results are also numerically calculated by the FLUENT software, whose detailed model based on finite volume method (FVM) can be seen in reference^53^. The good agreements can be found between any two of the experimental, numerical and analytical results, as shown in Figs. 3(b-c). It is indicated that the largest relative error is 3.39% between the present theory and experiment. Finally, it should be emphasized that the selection of *T*_∞_ plays an important role in the calculation of the advection heat flux, as shown in Eqs. (26a), (26b) and (31), especially for the engineering applications of complicated internal flows with the multiple outlets.

## Application

### The distributions of 3D heat flux vector

**Figure 4:**
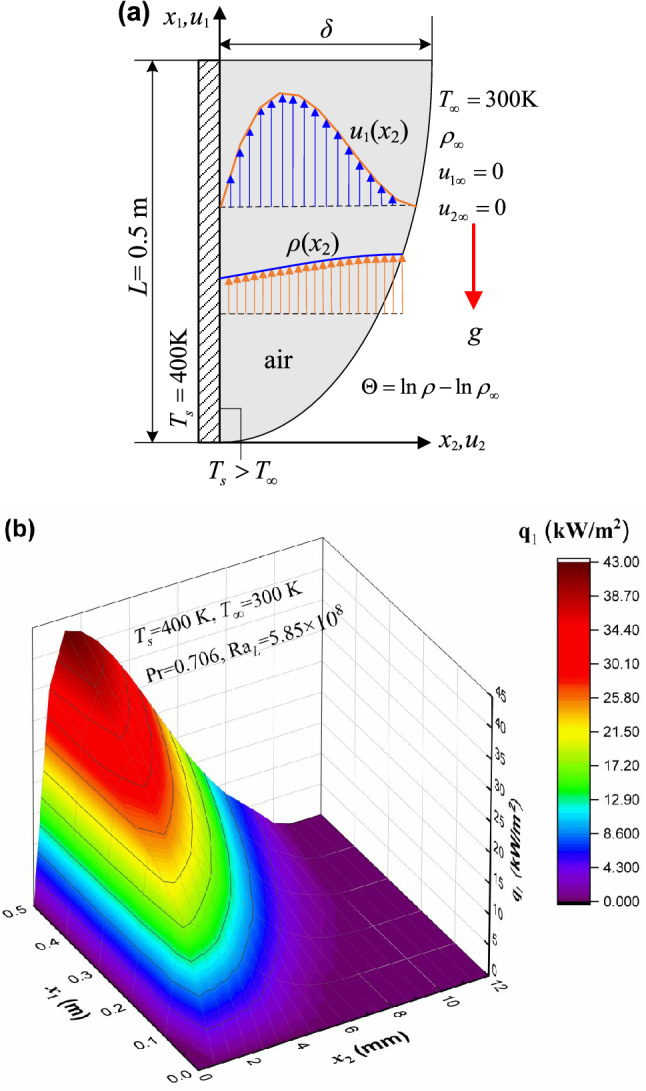

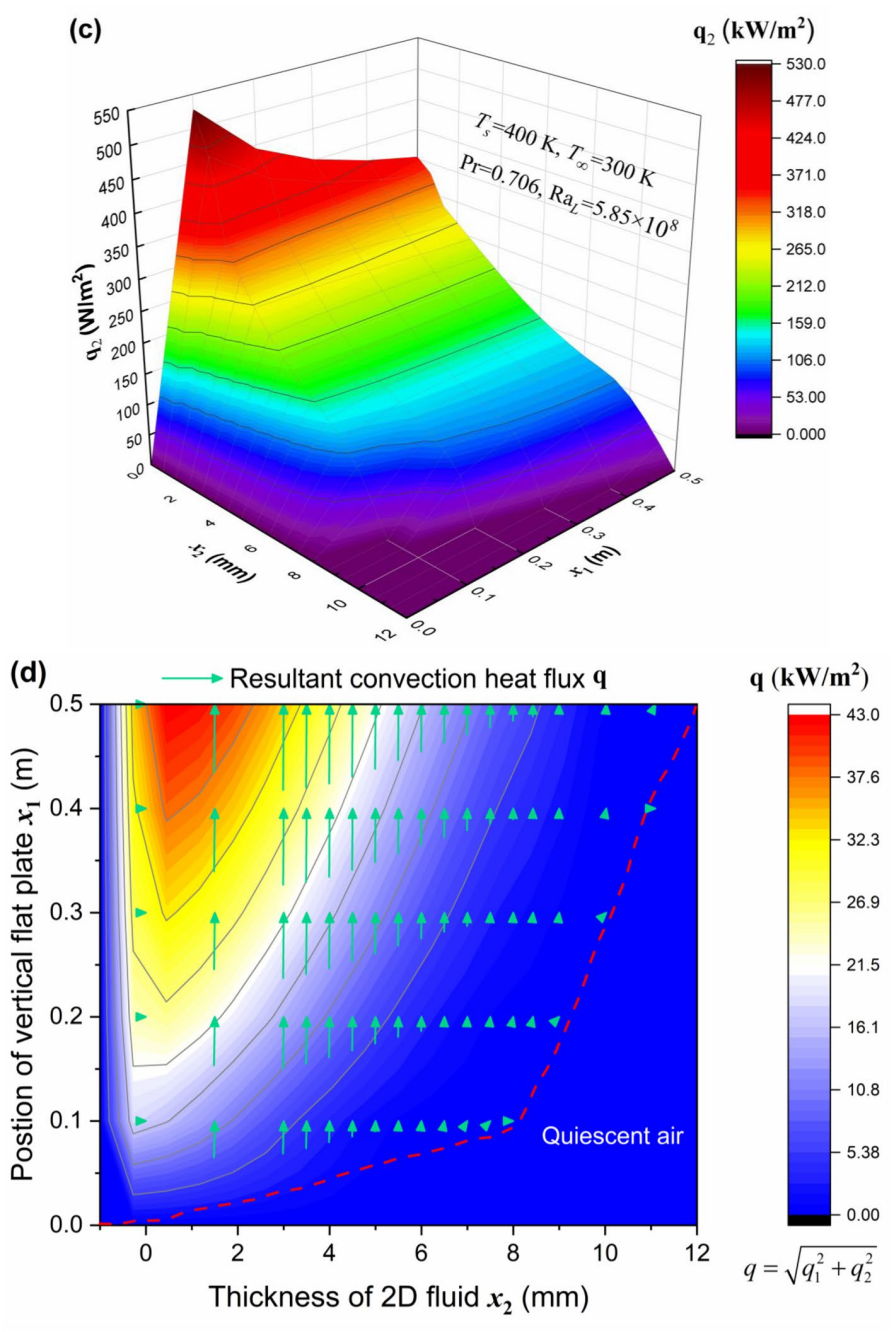
The spatial distributions of the heat flux vector of natural convection predicted by the present theory. (**a**) The steady laminar flowing air along the vertical flat plate surface under the constant surface temperature *T*_*s*_. Assuming the thickness of velocity boundary layer *δ* is the same as the thermal boundary layer. (**b**) The 3D theoretical profile of the streamwise convection heat flux **q**_1_ and that of (**c**) the wall-normal convection heat flux **q**_2_ along the plate length direction and across the fluid thickness direction, respectively, according to Eq. (36). Ra_*L*_ is the Rayleigh number at *x*_1_ = *L*, whose value is controlled to be less than 10^9^ to ensure the laminar flow obtained^21,22^. (**d**) The 2D vector map and contour lines of the resultant heat flux vector **q** for free convection obtained from the present theory. It is the resultant of its vectorial components **q**_1_ and **q**_2_, which is dominated by the main-stream-wise convective heat flux component **q**_1_.

The present convective heat transfer theory will be applied to the natural convection in external flows in this section, to indicate its capability of depicting the 3D convective heat flux vector (conductive heat flux plus advective heat flux) at any position in a compressible flow field, similar to Fourier’s law of heat conduction. We focus on natural convection flows bounded by a surface, and a classic example is associated with the boundary layer development on a heated vertical plate, as seen in Fig. 4(a). The plate is immersed in an extensive, quiescent air, and the air close to the plate is less dense than the fluid medium that is further removed owing to *T*_*s*_ > *T*_∞_^15^. The steady laminar flows along the vertical flat plate with impermeable surface and constant wall temperature *T*_*s*_ = 400 K are considered. Buoyancy forces therefore induce a natural convective boundary layer where the heated air rises vertically, entraining air from the quiescent region. The analytical profiles of velocity and density are obtained from the integral method^25^, in which the variable of logarithmic density difference ${\Theta } = ln \rho - ln {\rho_{\infty}}$ is employed. In particular, the velocity is zero as *x*_2_ → ∞, as well as at *x*_2_ = 0. The theoretical distributions of the vertical and transverse convective heat flux components along the plate length (*x*_1_ direction) and fluid thickness (*x*_2_ direction), and their resultant convective heat flux vector in Eq. (36) are rendered in Figs. 4(b–d), respectively, where the magnitude of resultant heat flux $q = \sqrt{q_1^2 + q_2^2}$. It is indicated that the heat advection and conduction respectively become the dominant heat transfer mechanism for the streamwise convective heat flux **q**_1_ [see Fig. 4(b)] and the wall-normal convective heat flux **q**_2_ [Fig. 4(c)]. The resultant heat flux vector diagram as well as the contour lines is plotted in Fig. 4(d). It is seen that **q**_1_ plays a dominant role in the energy transport process, and that **q** becomes higher as *x*_1_ increases and reaches its maximum at some region near the wall within the boundary layer. It is also found that there exists the steepest change of the resultant heat flux as well as that of density (or temperature) in the immediate neighborhood of the wall, as is consistent with the thermal boundary layer theory^25^. In accordance with the foregoing formulation (36), those profiles in Figs. 4(b–d) are, clearly, indicative of the fact that the present theory is capable of predicting the convection heat flux vector at arbitrary location in a compressible fluid stream quantitatively.

In conclusion, the unified formulae for the 3D heat flux vector of forced convection as well as natural convection are proposed for compressible laminar flows based on the first law of thermodynamics and others. It is found for a compressible single-phase fluid that the energy transport mechanism of advection is none other than the heat transfer owing to mass flow carried with enthalpy and mechanical energy by bulk fluid motion, driven by the temperature difference between the fluid temperature and the potential temperature caused by the adiabatic work done. The concept of potential temperature *T*_*ad*_ is introduced and developed to determine this adiabatic reference temperature. In order to describe the thermal driving force in the advection heat transfer process quantitatively, the reference temperature *T*_*ref*_ should be defined as the temperature at which zero heat transfer rate occurs. Therefore, *T*_*ref*_ in advection hear transfer for compressible flows is equal to *T*_*ad*_ [see Eq. (22)]. Apparently for incompressible flows, *T*_*ad*_ reduces to *T*_∞_ [see Eq. (24)], which refers to constant temperature at the inlet for internal flows and to the temperature at free-stream condition for external flows. We also found that an advective heat flux vector may be in the same or opposite direction as the fluid velocity vector, as is different from Fourier’s law of heat conduction. We have partially demonstrated the suggested convection heat flux formulae by employing the steady heat transfer experiment for an incompressible laminar flow inside the circular tube, but more experimental works for compressible flows are further needed to reveal the physical mechanism of convective heat transfer in the future. This work would bring fundamental insights into the physical mechanism of convection heat transfer and opens up a new arena for the design, calculation and thermal management of the 3D heat flux problems for single-phase compressible flows. Moreover, the proposed convective heat flux formulae also have the potential to be extended to apply to the turbulent convection heat transfer for compressible flows by adding the relevant fluctuation heat flux term^54^.

## Data Availability

The data that support the finding of this study are available from the corresponding author upon reasonable request.

## Abbreviations

*a* = *k*/(*ρc*_*p*_): Molecular thermal diffusivity of fluid, m^2^/s
*A*: Cross sectional area of inner surface within a fluid stream*,* m^2^
*c*_*p*_: Specific heat capacity at constant pressure, J/(kg•K)
*c*_*υ*_: Specific heat capacity at constant specific volume, J/(kg•K)
D(**∙**)/D*t*: Substantial derivative in rectangular coordinates
*e*: Specific internal energy, J/kg
*e*_*m*_: Specific mechanical energy, J/kg
*e*^0^ = *e* + *e*_*m*_: Combination of *e* and *e*_*m*_, J/kg
*e*_*t*_ = *e* + *pυ* + *e*_*m*_ = *h* + *e*_*m*_: Specific total energy for a flowing fluid, J/kg
${\dot E_{CV}}$: Rate of change of total energy, W
*g*: Acceleration of gravity, m/s^2^
*h* = *e* + *pυ*: Specific enthalpy, J/kg
*h*_*Newton*_: Cooling coefficient of Newton’s original rate equation, W/(m^2^•K)
*k*: Thermal conductivity, W/(m•K)
*L*: Total length of the experimental circular tube, m
*L*: Length of the vertical flat plate for natural convection analysis, m
$\dot m = \int_A {\rho {\mathbf{U}} \cdot {\mathbf{n}}} {\text{d}}A$: Mass flow rate, kg/s
**n**: Unit vector normal to the surface
*p*: Pressure, Pa
*P*: Constant surface heating power in experiment, W
*P*_*loss*_: Heat loss power, W
*P*_*net*_ = *P* − *P*_*loss*_: Net input of heat transfer rate, W
Pr = *ν*/*a*: Molecular Prandtl number
*q*_*i*_: Total convective heat flux component (*i* = 1, 2 and 3), W/m^2^
${\mathbf{q}} = \left\{ {{q_1},{q_2},{q_3}} \right\} = {{\mathbf{q}}_k} + {{\mathbf{q}}_u}$: Total (resultant) convective heat flux vector, W/m^2^
$\dot Q$: Heat transfer rate, W
*r*: Some arbitrary position within a fluid stream from the inlet, m
*R*: Radius of the experimental circular pipe, m
$R{a_{x_1}} = {{\beta g(T - {T_\infty })x_1^3} \mathord{\left/ {\vphantom {{\beta g(T - {T_\infty })x_1^3} {(\nu a)}}} \right. \kern-\nulldelimiterspace} {(\nu a)}}$: Rayleigh number for natural convection
*Re* = 2*u*_∞_*R*/*ν*: Reynolds number for tube flows
*t*: Time, s
*T*: Temperature of fluid, K
*T*_*ad*_: Potential temperature, K
*T*_*ref*_: Reference temperature, K
*T*_*s*_: Body’s uniform temperature at wall surfaces
*υ*≡1/*ρ*: Specific volume, m^3^/kg
*V*: Control volume, m^3^
*u*_*i*_: Fluid velocity component (*i* = 1, 2 and 3), m/s
*u*_∞_: Free-stream velocity for external flows, m/s
${\mathbf{U}} = \left\{ {{u_1},{u_2},{u_3}} \right\}$: Fluid velocity vector, m/s
*x*_*i*_: Space coordinate in the *i* direction in Cartesian system (*i* = 1, 2 and 3)

## Greek symbols

*β*: Volumetric coefficient of thermal expansion, 1/K
*κ*: Isothermal compressibility, 1/Pa
*ρ*≡1/*υ*: Density of fluid, kg/m^3^
*γ* = *c*_*p*_/*c*_*υ*_: Specific heat ratio
${\Theta } = \ln \rho - \ln {\rho_\infty }$: Difference of logarithmic density
∇: Gradient sign

## Subscripts

*ad*: Potential temperature condition
*CV*: Control volume
*i*: 1, 2 And 3 represent the streamwise, wall-normal and transverse directions in Cartesian system, respectively
*k*: Conduction condition in convective heat transfer
*m*: Mechanical energy
*p*: Potential temperature expressed by the variable of pressure
*r, φ*, *x*: Radial, circumferential and axial directions in cylindrical system, respectively
*ref*: Reference condition
*s*: Fluid condition at the wall surface
*u*: Advection condition in convective heat transfer
*υ*: Potential temperature expressed by the variable of specific volume
∞: Constant fluid condition at the inlet for internal flows or free-stream condition for external flows
